# Experimentally Achievable Accuracy Using a Digital Image Correlation Technique in measuring Small-Magnitude (<0.1%) Homogeneous Strain Fields

**DOI:** 10.3390/ma11050751

**Published:** 2018-05-08

**Authors:** Alice Acciaioli, Giacomo Lionello, Massimiliano Baleani

**Affiliations:** IRCCS—Istituto Ortopedico Rizzoli, Laboratorio di Tecnologia Medica, 40136 Bologna, Italy; alice.acciaioli@ior.it (A.A.); giacomo.lionello@ior.it (G.L.)

**Keywords:** digital image correlation, homogeneous strain, small deformation level, accuracy, precision, calcium phosphate cements

## Abstract

Measuring small-magnitude strain fields using a digital image correlation (DIC) technique is challenging, due to the noise-signal ratio in strain maps. Here, we determined the level of accuracy achievable in measuring small-magnitude (<0.1%) homogeneous strain fields. We investigated different sets of parameters for image processing and imaging pre-selection, based on single-image noise level. The trueness of DIC was assessed by comparison of Young’s modulus (E) and Poisson’s ratio (ν) with values obtained from strain gauge measurements. Repeatability was improved, on average, by 20–25% with experimentally-determined optimal parameters and image pre-selection. Despite this, the intra- and inter-specimen repeatability of strain gauge measurements was 5 and 2.5 times better than DIC, respectively. Moreover, although trueness was also improved, on average, by 30–45%, DIC consistently overestimated the two material parameters by 1.8% and 3.2% for E and ν, respectively. DIC is a suitable option to measure small-magnitude homogeneous strain fields, bearing in mind the limitations in achievable accuracy.

## 1. Introduction

Calcium phosphate cements (CPCs) are bone substitute materials used for tissue defects filling [1]. Although they should mimic the mechanical behavior of bone tissue, their mechanical properties are still far from optimal. In fact, CPCs are brittle [2,3,4], and the limited data available in the literature suggests that this material can only withstand small strain levels (range 0.1–0.2%) before failure [5,6]. Therefore, new CPC formulations are still under development [7].

CPC testing is performed on small specimens (typically up to 20 mm in their largest dimension) [8,9,10]. Accurately measuring strain values the material undergoes to during testing is useful to compare different formulations of CPCs regarding elastic response and toughness enhancement. However, due to CPC characteristics, this is a challenge. In fact, contact-type extensometers cannot be used, because the knife edges would damage the specimen surface. Conversely, strain gauge installation may affect CPC response because of the unavoidable penetration of cyanoacrylate adhesive into the pores, which are always present in the cement matrix [8,11,12]. Therefore, the alternatives are non-contact techniques, i.e., optical methods based on interferometric techniques, or digital image correlation (DIC).

Interferometric techniques may be highly accurate, but are not practical for large sample size studies. They also may be too sensitive to environmental conditions, or could be limited to measurements on quasi-static loading scenarios [13,14,15,16,17,18]. Conversely, the DIC technique can be easily implemented, as demonstrated by its wide range of applications [19,20,21,22,23]. Video extensometry, initially based on feature-based image registration techniques that is evolving into a real-time DIC [24], can measure high strain level with small errors. However, strain errors increases if the specimen’s dimensions decrease [25]. Moving on to DIC, experimental studies have defined effective procedures to calibrate DIC systems and/or to process the acquired images [26,27,28,29], thereby improving measurement accuracy. Unfortunately, although DIC has been demonstrated to be accurate in measuring medium-large strain levels [30,31,32,33,34,35,36], non negligible errors have been found when measuring small (<0.1% or 1000 microstrain) strain values [37,38,39,40], i.e., of the same order of magnitude of strain that CPCs can withstand before fracture. To the authors’ knowledge, there is only one report showing that the DIC technique has the potential to provide an accuracy level comparable to the strain gauge in measuring small strain values [41]. However, that study was carried out analyzing artificial images, i.e., in absence of experimental errors. Conversely, studies based on experimentally-acquired images suggest that the measurement of small strain values may be affected by errors with an order of magnitude of up to 10% [28,38,39,42,43,44].

The present study investigated the suitability of DIC for measuring small-magnitude, homogeneous strain field, by experimentally determining the accuracy achievable using DIC to measure Young’s modulus (E) and the Poisson’s ratio (ν) of a specimen subjected to strain levels smaller than 0.1%.

## 2. Materials and Methods

The strain gauge (SG) technique was chosen as a reference technique to determine the error in measuring E and ν using the DIC technique. Aluminum was used instead of CPCs, to avoid any problems related to the SG application (described in the introduction section), i.e., an undesired reinforcing effect on the porous brittle material. Five parallelepiped specimens (10 mm square cross section, 20 mm height) were machined from an AA1050 (maximum grain size 80 µm) square bar, in order to ensure material uniformity. Each specimen underwent two series of uniaxial compression tests.

**Figure 1:**
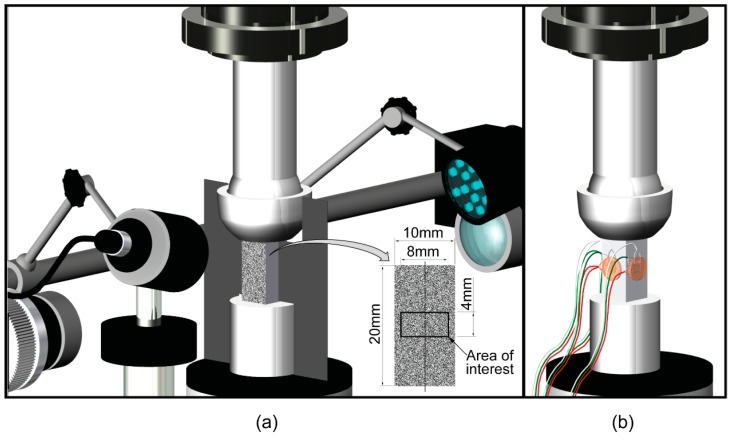
Scheme of the experimental setup used to measure surface strain. (**a**) Arrangement of the DIC system. The area of interest on the specimen surface is also shown. (**b**) Specimen instrumented with triaxial rosettes.

In the first series, a DIC system (Aramis 5M, GOM mbH, Braunschweig, Germany) was used to measure surface strain fields. Two digital cameras (2050 × 2448 pixels, TXG50i, Baumer Optronic GmbH, Radeberg, Germany), equipped with 2.8 FL/50 mm Titanar lenses and polarization filters (Schneider-Kreuznach, Bad Kreuznach, Germany) simultaneously monitored two opposite sides of the specimen at 15 Hz (Figure 1a).

Both cameras were mounted onto a rigid support integrated into the testing machine frame and oriented in order to have the specimen surface centered in the view, while assuring a perpendicularity error smaller than 0.5 degrees. The camera-specimen distance was adjusted to the minimum focusing distance—about 150 mm from the polarizing filter placed in front of the camera lens to the specimen surface—obtaining a 12.6 × 15.0 mm measuring window. The resulting pixel size was 0.006 mm. A smaller pixel size, experimentally achievable with higher focal length lens, would have required a smaller dot size—determining the need to replace the airbrushing procedure (see below)—and would have reduced the subset dimension into millimeters (see below), thus introducing potential noise, due to the local strain gradient that occurs across grain boundaries. Speckle patterns (black dots on a white background) were previously created on two opposite surfaces of the specimen using an airbrush (Iwata HP-CH, 0.3 mm nozzle, Anest Iwata Europe S.r.l., Torino, Italy). Airbrush settings (air pressure = 3 bar; paint reduction by volume = 40%; airflow at the nozzle = 2 screw turns; needle travel length = 3 screw turns; spraying distance about 15 cm) were chosen using an internal airbrush-specific algorithm [45], in order to achieve an optimal speckle size of 3–5 pixels [46,47]. A trained operator (A.A.) created all patterns trying to achieve a coverage factor falling within the range of 42–50%, in order to minimise strain noise [48]. Average speckle size and coverage factor, calculated using the technique proposed by Lecompte et al. [49] and Shih [50], were 4.3 pixels (mean value range: 4.1–4.5 pixels) and 49% (range 47–50%) respectively. Images were acquired under the best achievable experimental conditions, i.e., at the smallest lens aperture (1/16, to get a sufficient depth of field), using the maximum exposure time (56 ms, due to the frame rate set to 15 Hz), while maximizing uniform lighting conditions over the entire area of interest (a white-light led was placed at about 80 mm from the specimen surface, the minimum allowable distance without interference of the 9W-led body with the field of view while maintaining the lamp heat-sink above and behind the camera lens). 2D calibration of each camera was carried out before each test-session by using a cubic-shaped 15 × 12 mm panel, following the manufacturer’s recommendations [28].

An 8 by 4 mm rectangular area in the center of the specimen surface (Figure 1a) was selected as the area of interest, to exclude a local effect due to end effects. Preliminarily, the interaction between the subset size, step size, and strain window was investigated to select optimal parameters for image processing. The theoretically-set optimal subset size was calculated as three times the sum of the mean of the speckle size and distance (calculated values fell in the range 25 × 25–28 × 28 pixels), rounded to the nearest multiple of ten. Therefore, a subset size of 30 × 30 pixels was chosen. The step size was set at 1, 3, 8 and 15 pixels.

**Figure 2:**
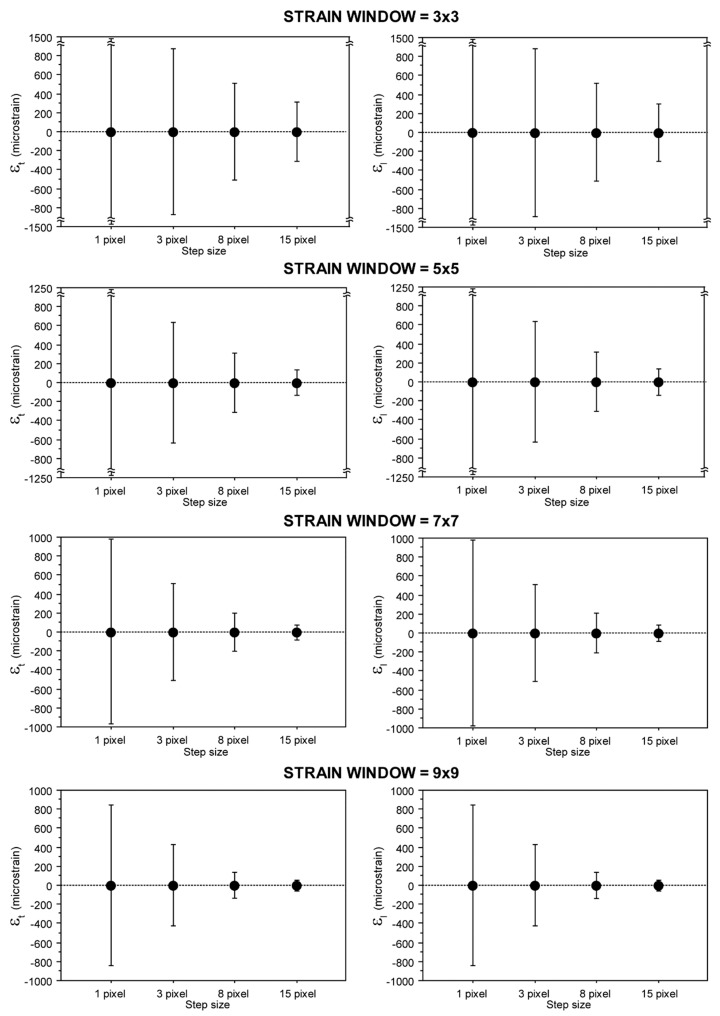
Errors in a zero-strain map achieved using a subset size of 30 × 30 pixels with different overlap values and strain window dimensions (error bar = standard deviation).

Image processing was carried out using a dedicated software (Aramis V 6.3.0, GOM mbH, Braunschwieg, Germany). Surface strain in a measurement point, i.e., the center of a subset, was calculated using a square grid containing the center points of N × N neighboring subsets [51,52], hereinafter referred to as the strain window. The 2D deformation gradient tensor was calculated solving the system of equation, based on a first-order shape function, by the least square method [53,54]. The strain window was increased, starting from 3 × 3 subset, i.e., a field of center points of 3 × 3 subset, and increasing in steps of 2 subsets, up the maximum dimension of 9 × 9. Since increasing the step size decreased the noise in zero-strain maps (Figure 2), and the processing time without losing independent data, a step size of 15 pixels was chosen, i.e., an overlap ratio between neighboring subsets of 50% [55,56]. Similarly, a 9 × 9 strain window dimension was selected, because the noise decreased in zero-strain maps by increasing the strain window (Figure 2) [57]. Therefore the first image processing was carried out using a subset size of 30 × 30 pixels, a 50% overlap ratio and a 9 × 9 strain window.

**Figure 3:**
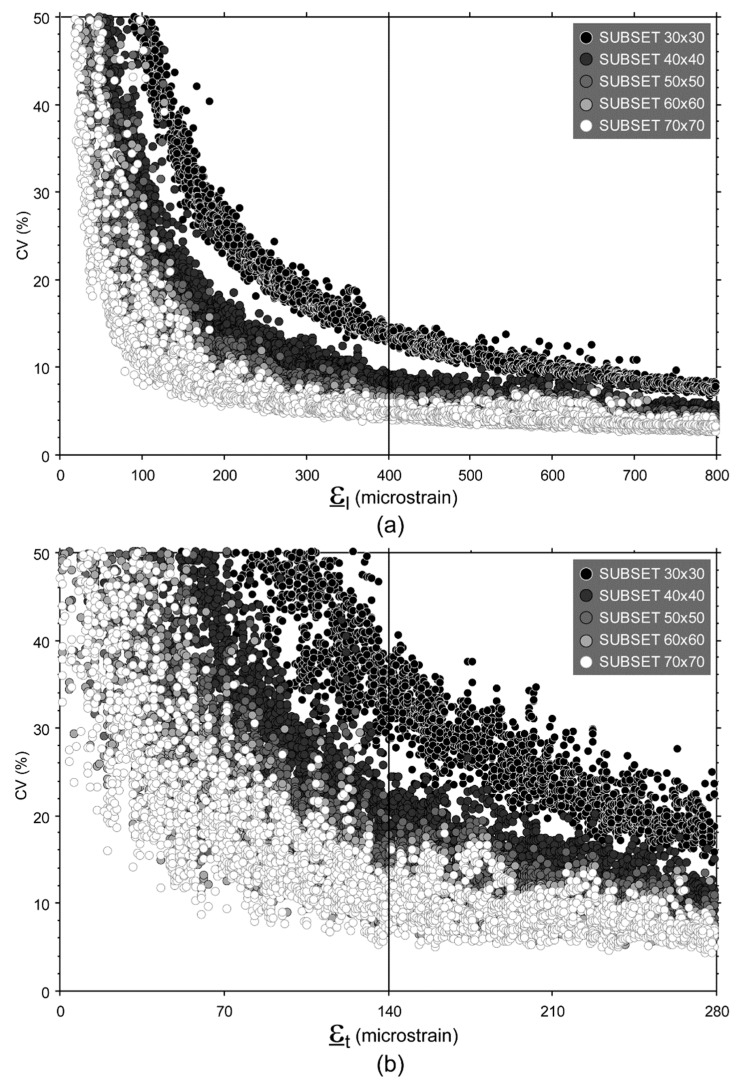
Representation of CV values calculated on the same image datasets changing the subset size from 30 × 30 to 70 × 70 pixels. (**a**) ε_l_ values; (**b**) ε_t_ values. Note: Each circle represents the CV of all strain values (ε) measured on the area of interest of a couple of images simultaneously acquired on both sides of the specimen.

A second image processing was carried out using the same parameters, except for the subset size, which was set at 60 × 60 pixels. This size was experimentally determined starting from the previous (30 × 30 pixels), and increasing it in steps of 10 pixels. The selection of the experimentally-determined optimal subset size to be used for image processing was based on the values of the coefficient of variation (CV, i.e., the ratio of the standard deviation to the mean, expressed in percent) of the principal strain values calculated for pooled data obtained from each couple of images. Indeed, CV values, which should theoretically be zero, are affected by both experimental and processing errors; the latter decreases by increasing the subset size. Since no further significant reduction was found when passing from pixel sizes 60 × 60 to 70 × 70, the 60 × 60 dimension was selected as the experimentally determined optimal subset size, as mentioned above (Figure 3, see also Supplementary Materials, Figure S1).

**Figure 4:**
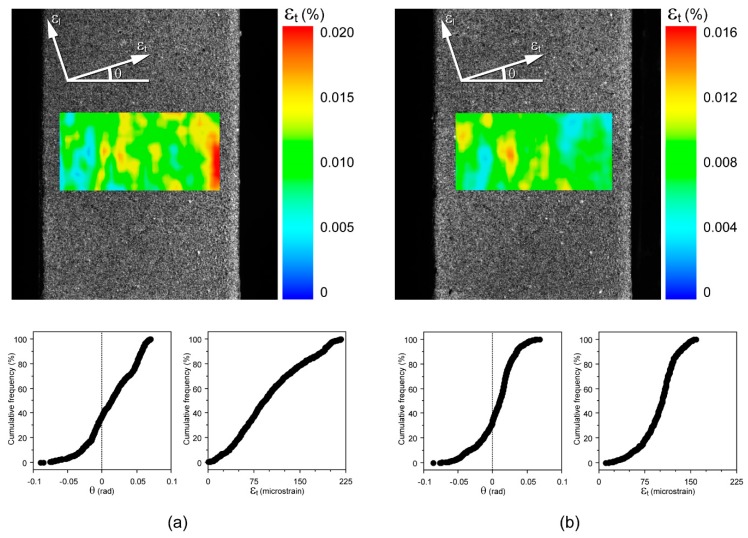
Two strain maps, with a cumulative distribution of θ and ε_t_ values, calculated for the same ε_l_ value (ε_l_ = 280 microstrain) on the same specimen surface during two different test-sessions. (**a**) 3SD_θ_ > 0.1 rad: this image, and the image simultaneously acquired on the opposite surface of the specimen, must be discarded; (**b**) 3SD_θ_ ≤ 0.1 rad: this image is accepted if θ distribution determined for the image simultaneously acquired on the opposite surface of the specimen fulfils the same requirement.

A third image processing step was carried out using the previous parameters and an image pre-selection. Since noise on the strain map determines angle fluctuation of principal compressive (or longitudinal strain ε_l_) and tensile strain (or transverse strain ε_t_) direction, image pre-selection was based on the distribution of angle fluctuation: an image, and the image simultaneously acquired on the opposite surface of the specimen, was automatically discarded when three times the standard deviation of θ values (3SD_θ_) was greater than 0.1 rad, where θ was the angle between local ε_t_ and the horizontal direction (Figure 4).

For all the three image processing, the average values (indicated as ε) of ε_l_ and ε_t_ were calculated over the two areas of interest which were simultaneously acquired by the two cameras.

In the second series, triaxial rosettes with pre-attached lead wire (UFRA-3-350, Tokyo Sokki Kenkyuio Co, Tokyo, Japan) were used to measure strain reference values; this was necessary to determine the accuracy of the DIC technique (Figure 1b). After having carefully prepared the surface (Vishay Precision Group, 2014), a rosette was attached in the center of all four sides of the specimen surface (Figure 1b). Strain data were acquired at 100 Hz, using a multichannel data logger (System 6000, Vishay Precision Group, Raileigh, NC, USA), and processed to determine the principal directions and principal strains using a dedicated software (StrainSmart V4.31, Vishay Precision Group, Raileigh, NC, USA). The average values (ε) of ε_l_ and ε_t_ simultaneously acquired by the four rosettes were calculated.

Both test series were performed at a constant displacement rate of 0.1 mm/min. Specimens were mounted onto a fixed platen placed onto a 10 kN load cell and loaded through an unlocked spherical seat platen fixed to the actuator of the testing machine (Mod.8502, Instron, Norwood, MA, USA), allowing alignment to the specimen end surface. A customized jig was used in order to align the specimen to the spherical seat, while maintaining the two patterned surfaces orthogonal to the DIC cameras. An initial preload (about 20 N) was applied to maintain the position of the specimen before removing the customized jig. All tests were limited to −5.6 kN, in order to reach a maximum compressive strain value just above a target value of 800 microstrain (i.e., 0.08%). The minimum compressive strain value was chosen considering the image processing errors. Indeed, referring to the experimentally-determined optimal subset size 60 × 60, CV values started to increase exponentially for ε_t_ values falling below 140 microstrain (Figure 3). Therefore this value, which corresponds to 400 microstrain in term of ε_l_, was set as minimum strain value in calculating E and ν.

Each test-session of five specimens was repeated five times after dismounting and remounting the whole setup. For each test repetition, E and ν were calculated using ε_l_ and ε_t_, measured as described above using both techniques. ε_l_ values were limited to a range of 400–800 microstrain for the aforementioned reasons. The E value was determined as the slope of the stress-ε_l_ curve. The ν value was calculated as the mean value of all the absolute ratios of ε_t_ to ε_l_. Inter- and intra-specimen repeatability was expressed as CV, calculated for five repetitions carried out on the same specimen and for each set of five specimens respectively. DIC trueness was assessed by analyzing differences between E and ν mean values, which were determined using DIC and SG techniques on each specimen, by means of a paired t-test.

Finally, on the basis of the achieved results (see below), the third image processing was used to determine the elastic properties of a CPC prototype formulation. The formulation was optimized for 3D plotting, i.e., the paste allowed extrusion from a thin needle and assured printed shape stability. Ten parallelepiped specimens (10 mm square cross section, 20 mm height) were printed in air and immersed in deionized water for two weeks to achieve full setting. CPC specimens underwent monotonic compressive tests performed at a constant displacement rate of 0.1 mm/min. E and ν were calculated limiting ε_l_ to the range of 400–800 microstrain for the aforementioned reasons. 

## 3. Results

Noise in zero strain readings was negligible for SG; in all cases, measured values were smaller than 4 microstrain. Conversely, although ε_t_ and ε_l_ values calculated using DIC technique were smaller than 10 microstrain, ε standard deviation was 10–20 microstrain, with peak values up to 70 microstrain.

**Figure 5:**
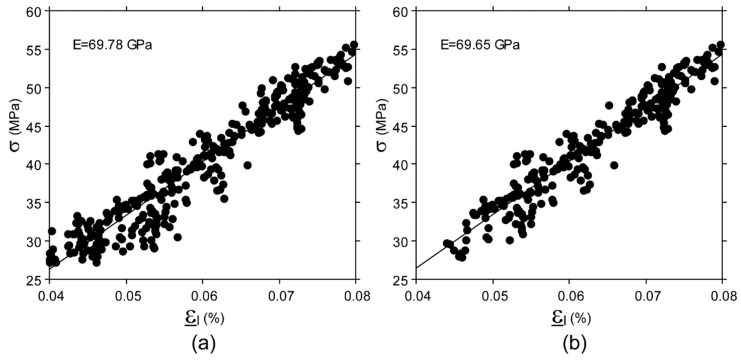
E value determined for one specimen. (**a**) Regression slope obtained using a subset size of 60 × 60 pixels. (**b**) Regression slope obtained using a subset size of 60 × 60 pixels and image pre-selection.

**Figure 6:**
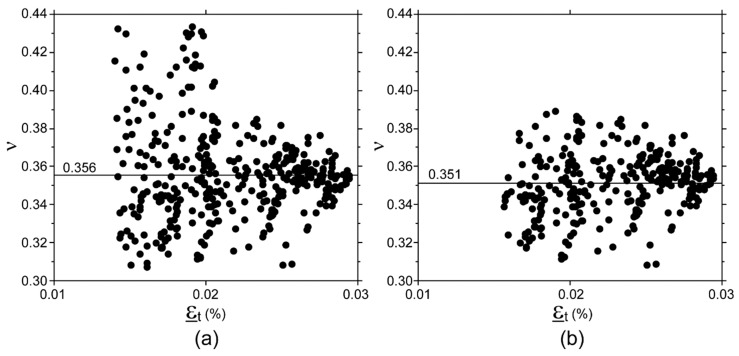
ν mean value determined for one specimen. (**a**) Data obtained using a subset size of 60 × 60 pixels. (**b**) Data obtained using a subset size of 60 × 60 pixels and image pre-selection. Note 1: The 140 microstrain threshold to ε_t_ had already been applied before calculating ν values reported in this graph. Note 2: Interchanging ε_l_ to ε_t_ in *x*-axis would: (i) scale the *x*-axis by a factor of 1/0.35 (ii) cause a small horizontal drift of each point, the entity and direction of which would depend on the difference between the current value and 0.35; no changes would occur in ν mean values.

Approximately, 360 images were captured by each camera during a test against about 2400 triplets of data acquired from each rosette. Image pre-selection was used in the third processing procedure for DIC data. This process discarded, on average, 12% of images (range 0–35%). Discarded images were generally acquired in the first part of the test, i.e., where ε_t_ values calculated using DIC fell into the lower part of ε_t_ range. An example of the effect of image pre-selection on collected data is show in Figure 5 and Figure 6.

Intra- and inter-specimen repeatability values, calculated for the two techniques, are summarized in Table 1 and Table 2 for E and ν values respectively. The three CV values reported for DIC technique refer to the three different image processing procedures. Intra- and inter-specimen repeatability of DIC were comparable. Conversely, inter-specimen repeatability of SG was double that of intra-specimen. In general, both techniques were more precise for determining E values. However, regardless of the measured parameter, the intra- and inter-specimen repeatability of SG measurements was better than DIC by about 5 and 2.5 times respectively. An improvement in DIC repeatability was found by increasing the subset size, and by adding image pre-selection. This improvement was more noticeable when looking at the worst CV values calculated for ν values.

**Figure 7:**
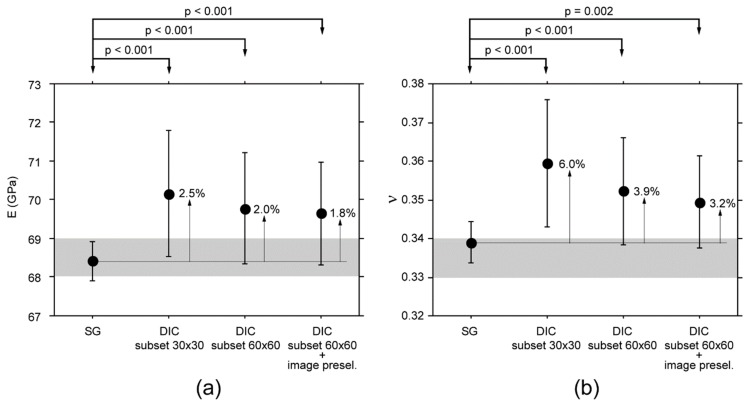
(**a**) E and (**b**) ν mean values, and relative standard deviations, determined using SG and DIC technique. The grey bands represent the range of E and ν values for AA1050, as reported in the literature. Note: The total sample size is 25, with 5 repeated measurements taken on the same specimen.

The trueness of DIC is shown in Figure 7, where values experimentally determined using SGs were used as reference. Although the bias decreased by increasing the subset size and adding image pre-selection, the difference was statistically significant in all three comparisons.

The pilot study on a CPC prototype formulation was successfully carried-out using the described experimental procedure. Indeed, elastic mechanical properties were determined for all ten tested specimens. E and ν were 9.0 ± 1.2 GPa and 0.22 ± 0.04 respectively.

## 4. Discussion

This study shows that DIC can measure small-magnitude (<0.1%) homogeneous strain fields with satisfactory accuracy, as demonstrated by the good agreement between E and ν values determined using SG (reference values) and DIC technique.

Although the DIC technique is extensively used to investigate material response to loads, these results were not foreseeable because, in general, published studies measured surface strain values with magnitudes of 1% or greater, both at macro and microscopic scales, [23,25,35,36,42,58,59,60] and reported error values of the same order of magnitude of the strain range as those investigated in the present study [28,39,42,43,44,61]. Indeed, the above-mentioned accuracy was achieved by minimizing the noise level, by (i) measuring each strain value (ε) on a 60 × 60 pixels subset, using a 50% overlap ratio (step size 30 pixel) and a 9 × 9 strain window, meaning that each strain value was calculated on a squared area of 300 × 300 pixels, i.e., 1.8 × 1.8 mm, and (ii) averaging all the strain values measured over an area of 64 mm^2^ to calculate the ε_l_ and ε_t_ value for each couple of images simultaneously acquired, i.e., averaging about 900 ε values. Despite all this, some level of noise in ε_l_ and ε_t_ values, of up to 40 microstrain (in agreement with previous findings [62]), still remains, as shown by the dispersion of ν values in Figure 6. It has been widely demonstrated that the overlap ratio (or step size), subset size, and strain window dimension (or computation size) in image processing impact the DIC accuracy. These parameters were optimized in the present study for the described setup. Indeed, the chosen overlap ratio fell into the optimal range recommended in the literature, i.e., 50–75% [52,63,64]. A greater overlap ratio, i.e., smaller step size, would produce oversampled data, and increase noise on the strain field [48,65]. Similarly, a subset size greater than 60 × 60 pixels would not further reduce the residual error. It is acknowledged that increasing the subset size will increase the accuracy with which displacements are determined [49,66,67]. Indeed, CVs of ε values decrease as the subset size increases from 30 × 30 to 60 × 60, as shown in Figure 3. However, beyond a 60 × 60 pixel size, no further improvement was found; this is in agreement with previous studies showing that there is a plateau in noise reduction [25,42,66,67]. It must be acknowledged that subset sizes used in the literature are generally smaller than 60 × 60 pixels [30,31,68,69]. However, those studies investigated strain levels greater than 1%. Indeed, trends observed in Figure 3 suggest that subset size smaller than 60 × 60 become appropriate for measuring strain levels greater than 0.08%, thus increasing the ability of DIC to capture strain gradients. However when strain levels smaller that 0.1% are investigated, the optimal subset size increases [41,70]. It is noteworthy that CV values do not converge to zero. It seems unlikely that this noise is due to strain localization across grain boundaries, since the subset dimension, in millimeters, was at least one order of magnitude greater than the grain dimension. In other words, we assumed that the aluminum surface deformation appeared homogeneous at subset scale level. Noise in DIC measurements contributes to a residual trend. However, the actual strain distribution was non-uniform over the cross section, due to undesired off-axis loading on the specimen. In fact, although specimen alignment was performed carefully, a misalignment error of up to 0.1 mm may have occurred, which contributes up to 6.5% to residual CV value. The DIC setup (i.e., two cameras monitoring simultaneously two opposite side of the specimen), as well as the four-rosette configuration, was chosen in order to compensate for the misalignment error, and to determine accurate E value. Referring to the strain window dimension, a large value (9 × 9) was used, i.e., each ε value was calculated over a quite large area, as explained above. This choice decreases the ability of DIC to capture strain gradients, and therefore to accurately monitor what happens in proximity of any nucleating crack, which is irrelevant in the homogeneous strain field. Greater strain window dimensions might further lightly smooth ε values, but would not change the ε value calculated over the entire area of interest, and ultimately, would not change the results.

Other known sources of error are due to inaccuracies in preparing the specimen surface (speckle pattern), or in setting up the experimental model (loading condition, lens alignment, lighting, etc.). Special attention was paid in the specimen preparation. The speckle pattern was created by spraying black dots on the specimen surface following a previously developed procedure [45]. This involved the use of an airbrush-specific algorithm, which allows a trained operator to achieve the desired dot size of 3–5 pixels, and a coverage factor closer to 50%. On the basis of the data available in the literature [42,47,48,65,71], the achieved pattern should minimize the correlation error. In addition, the experimental model was set up attempting to achieve the best operating conditions. Additionally, the experimental setup minimized the error of perpendicularity between camera and specimen surface, decreasing the out-of-plane motion. Indeed, residual errors in calculating Poisson’s ratio determined using the DIC technique, are comparable to those reported in the literature using the DIC 3D setup [72], which is unaffected by out-of-plane motion, without the need to manipulate DIC data. Finally, another source of error was deleted by simultaneously monitoring two opposite sides, in order to compensate for the problem of a non-axial-load applied on the specimen due to small misalignment between specimen axis and spherical seat. However, other errors are operator dependent (e.g., focusing camera lenses, lighting homogeneously both specimen surfaces). Despite the fact that the same trained operator carried out all test-sessions, the repeatability in achieving the experimental optimal conditions for the investigated strain range appeared to be a problem. However, it must be acknowledged that other sources of errors, such as thermal noise or CCD sensor noise [73] may have affected strain measurements. Whatever the cause, when experimental “sub-optimal” conditions occurred, image pre-selection played a role in reducing error and slightly improving DIC accuracy. In fact, image pre-selection discarded several images in all but one test session. The image pre-selection criterion is based on a priori knowledge of strain directions. It deletes all and only images that are noisy, which is more effective than data filtering, slightly improving both DIC trueness and precision in determining E and ν values (although a residual bias and a quite large dispersion still exists). Dispersion values can be ascribed to specimen inhomogeneity only for a small part. Indeed, apart from the fact that specimens were machined from the same bar, in order to minimize material inhomogeneity due to processing route, the inter-specimen repeatability achieved using SG was excellent. It could be argued that the E and ν values determined using SG are not true values, and therefore, that it is not possible to conclude that DIC overestimates the two mechanical parameters. However, although SG data are affected by some degree of uncertainty, the E and ν values determined using SG are in agreement with those reported in the literature for AA1050. This observation indirectly supports the aforementioned conclusion.

Other known sources of error are the quality of DIC hardware [28,74,75], and the correlation algorithm [34,37,54,74,76,77]. However, it was out of the scope of this study to compare different hardware or algorithms. Therefore, one commercial system was used to carry out the experimental series; this is a major limitation of this work. In fact, it cannot be excluded that different hardware components or different sub-pixel registration algorithms might have further improved the accuracy and precision achieved in the present study. However, the described procedure appeared suitable to determine CPC elastic mechanical properties, whose accuracy is a key for the reliability of numerical models of CPC 3D structures.

## 5. Conclusions

DIC can be practical and effective in measuring small-magnitude (<0.1%) homogeneous strain fields. The experimentally-determined optimal parameters for image processing, including the identification of the optimal subset size for the actual speckle pattern, and image pre-selection based on strain direction, can minimize the noise level. Under these conditions, a trueness better than 2% and 4% in measuring E and ν can be achieved, keeping in mind that both parameters are overestimated. However, DIC repeatability must be taken into account when calculating the sample size.

## Figures and Tables

**Table 1:** Intra- and inter-specimen repeatability for E values. The mean and the worst (maximum) CV values are reported.

Young’s Modulus (E)	Strain Gauge Mean (Worst)	DIC Subset 30 × 30 Mean (Worst)	DIC Subset 60 × 60 Mean (Worst)	DIC Subset 60 × 60 + Image pre-Selec. Mean (Worst)
Intra-specimen repeatability	0.4% (0.7%)	2.2% (2.4%)	1.9% (2.2%)	1.8% (2.0%)
Inter-specimen repeatability	0.8% (1.0%)	2.3% (2.9%)	2.0% (2.7%)	1.9% (2.4%)

**Table 2:** Intra- and inter-specimen repeatability for ν values. The mean and the worst (maximum) CV values are reported.

Poisson’s Ratio (ν)	Strain Gauge Mean (Worst)	DIC Subset 30 × 30 Mean (Worst)	DIC Subset 60 × 60 Mean (Worst)	DIC Subset 60 × 60 + Image Pre-Selec. Mean (Worst)
Intra-specimen repeatability	0.8% (1.2%)	4.4% (6.4%)	3.8% (5.4%)	3.3% (4.4%)
Inter-specimen repeatability	1.6% (2.2%)	4.3% (6.3%)	3.8% (5.5%)	3.5% (4.9%)

## Supplementary Materials

The following are available online at http://www.mdpi.com/1996-1944/11/5/751/s1, Figure S1: Errors in a zero-strain map achieved using a subset size of 60 × 60 pixels with different overlap values and strain window dimensions (error bar = standard deviation).
