# Supplementary material for: Experimentally Achievable Accuracy Using a Digital Image Correlation Technique in measuring Small-Magnitude (<0.1%) Homogeneous Strain Fields
Source: Materials (Basel). 2018 May 8;11(5):751. doi: 10.3390/ma11050751 (PMC5978128; doi:10.3390/ma11050751)
Supplement: Supplementary file 1 [file materials-11-00751-s001.pdf]

materials

Supplementary Materials

Experimentally Achievable Accuracy Using a Digital Image Correlation Technique in measuring Small-Magnitude (<0.1%) Homogeneous Strain Fields

Alice Acciaioli, Giacomo Lionello and Massimiliano Baleani *

IRCCS—Istituto Ortopedico Rizzoli, Laboratorio di Tecnologia Medica, 40136 Bologna, Italy; alice.acciaioli@ior.it (A.A.); giacomo.lionello@ior.it (G.L.)

* Correspondence: baleani@tecno.ior.it; Tel.: +39-051-636-6865

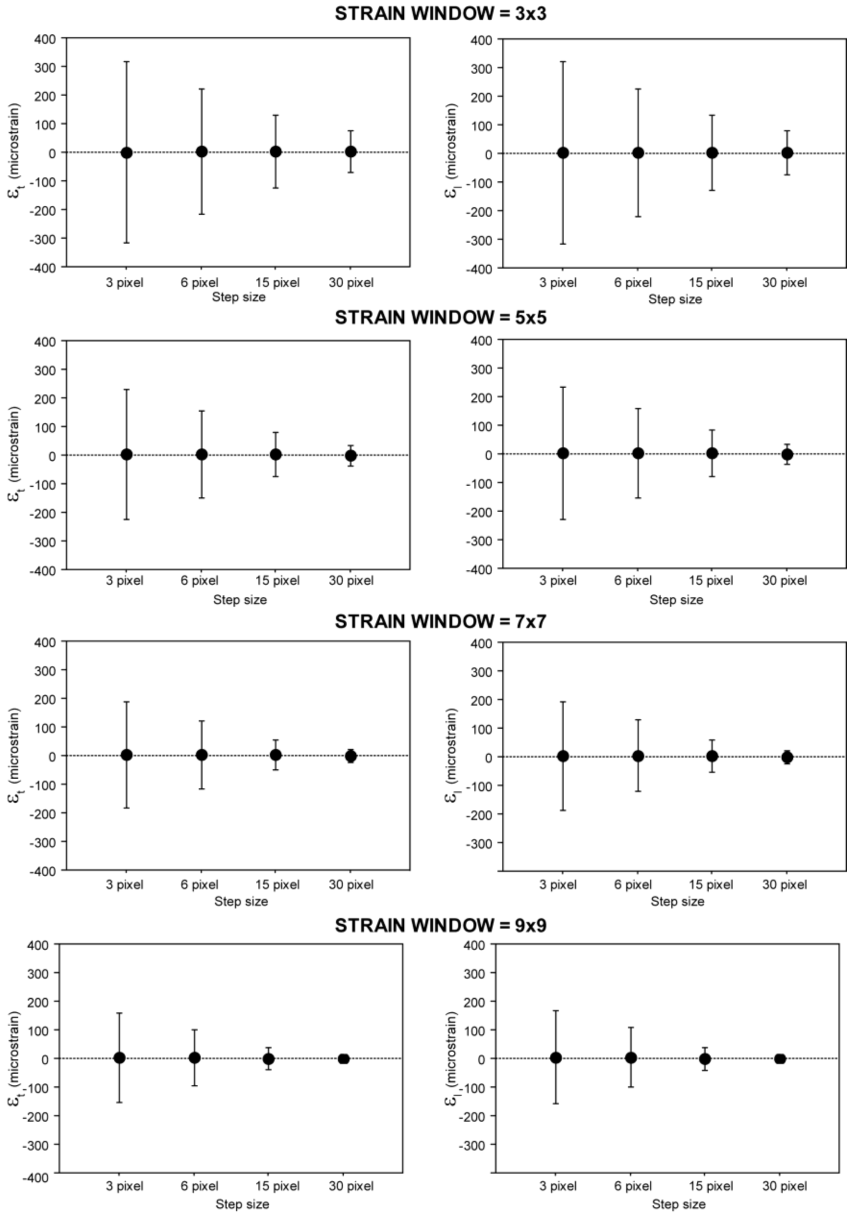

Figure S1. Errors in a zero-strain map achieved using a subset size of 60 × 60 pixels with different overlap values and strain window dimensions (error bar = standard deviation).
